# Nutritional status modulates box C/D snoRNP biogenesis by regulated subcellular relocalization of the R2TP complex

**DOI:** 10.1186/s13059-014-0404-4

**Published:** 2014-07-25

**Authors:** Yoshito Kakihara, Taras Makhnevych, Liang Zhao, Weiwen Tang, Walid A Houry

**Affiliations:** 1 King’s College Circle, Medical Sciences Building, Department of Biochemistry, University of Toronto, Toronto, Ontario M5S 1A8 Canada

## Abstract

**Background:**

Box C/D snoRNPs, which are typically composed of box C/D snoRNA and the four core protein components Nop1, Nop56, Nop58, and Snu13, play an essential role in the modification and processing of pre-ribosomal RNA. The highly conserved R2TP complex, comprising the proteins Rvb1, Rvb2, Tah1, and Pih1, has been shown to be required for box C/D snoRNP biogenesis and assembly; however, the molecular basis of R2TP chaperone-like activity is not yet known.

**Results:**

Here, we describe an unexpected finding in which the activity of the R2TP complex is required for Nop58 protein stability and is controlled by the dynamic subcellular redistribution of the complex in response to growth conditions and nutrient availability. In growing cells, the complex localizes to the nucleus and interacts with box C/D snoRNPs. This interaction is significantly reduced in poorly growing cells as R2TP predominantly relocalizes to the cytoplasm. The R2TP-snoRNP interaction is mainly mediated by Pih1.

**Conclusions:**

The R2TP complex exerts a novel regulation on box C/D snoRNP biogenesis that affects their assembly and consequently pre-rRNA maturation in response to different growth conditions.

**Electronic supplementary material:**

The online version of this article (doi:10.1186/s13059-014-0404-4) contains supplementary material, which is available to authorized users.

## Background

The ribosome is a highly complex machinery dedicated to protein synthesis. Its biogenesis involves a large number of protein and RNA factors consuming a considerable portion of the cellular energy [1,2]. Eukaryotic cells require more than 70 ribosomal proteins (r-proteins) and four distinct rRNAs (25S/28S, 18S, 5.8S, and 5S) for ribosome formation. In yeast, three of the four rRNAs (25S, 18S, and 5.8S) are generated from a 35S pre-rRNA, which has to be extensively processed and modified into the mature rRNAs. The processing of pre-rRNA is carried out by a large number of small nucleolar ribonucleoproteins (snoRNPs) and numerous non-ribosomal trans-acting factors such as endo- and exonucleases and RNA helicases [1,3-5].

There are two major classes of snoRNPs, box C/D and box H/ACA, that are responsible for 2′-O-methylation and pseudouridylation of pre-rRNA, respectively [1,6]. The box C/D snoRNPs consist of box C/D snoRNAs and four core proteins: Snu13, Nop1, Nop56, and Nop58. The snoRNA guides the snoRNP complex to the methylation site on the pre-rRNA by base pairing [7,8]. Nop1 (fibrillarin in vertebrates), which contains the signature motif of S-adenosyl-methionine RNA methyltransferase, catalyzes the methylation of the 2′-hydroxyl group of the target ribose on the pre-rRNA [9,10].

It has been proposed that box C/D snoRNPs assemble hierarchically. In vertebrates, the formation of box C/D snoRNA-15.5 K (Snu13 ortholog) complex is required for the assembly of the other core proteins with the snoRNP [11]. Similarly, stepwise association has been observed in archaea beginning with the interaction between box C/D sRNA and L7Ae (Snu13 homolog), followed by the binding of Nop5 (Nop56/Nop58 homolog) and then the recruitment of fibrillarin into the complex [10]. A number of protein factors are required for the assembly of snoRNPs. These factors include the R2TP complex, Hsp90, Bcd1, Rsa1, and Srp40/Nopp140 in yeast [12-15]. Other additional factors in higher eukaryotes include TAF9, PHAX, CRM1, CBC, Ran, and Snurportin1 [16-20].

The R2TP complex was discovered by our group in yeast *S. cerevisiae* [21]; it is composed of four proteins: Rvb1, Rvb2, Tah1, and Pih1. The complex is highly conserved from yeast to mammals [22]. Rvb1 and Rvb2 are members of AAA + (ATPase associated with diverse cellular activities) superfamily and, in addition to snoRNP assembly, are involved in many other critical cellular processes such as chromatin remodeling, DNA replication, DNA damage repair, transcription, and telomerase assembly [23,24]. Pih1 (also known as Nop17) was initially isolated as a Nop58 interacting protein in yeast [25] and, subsequently, shown to be part of the R2TP complex and to interact with Hsp90 [14,21]. Tah1 contains two tetratricopeptide repeat (TPR) motifs and acts as a co-factor for Hsp90 to stabilize Pih1 [14,26]. We and others have shown that the R2TP complex functions as a snoRNP assembly factor in yeast [14] and mammalian cells [13]. More recently, the complex has been found to be also required for other cellular processes in mammals such as assembly of RNA polymerase II [27,28], phosphatidylinositol 3-kinase-related kinases (PIKKs) signaling pathway [29], and apoptosis [30-32].

Here, we show that the R2TP complex strongly interacts with unassembled Nop58, that the function of R2TP in box C/D snoRNP biogenesis is mediated by the interaction of Pih1 with Nop58, and that the R2TP complex stabilizes Nop58. Importantly, the R2TP-Nop58 interaction was found to be growth-phase or nutrient-condition dependent. Also, we uncovered a shift in the cellular distribution of R2TP proteins between the cytoplasm and the nucleus based on the growth phase of the cell and nutrient condition. Our data suggest that this dynamic subcellular relocation of the R2TP complex could regulate its function, which, subsequently, affects box C/D snoRNP assembly and, hence, modulates pre-rRNA processing. Our findings describe a novel regulatory mechanism for box C/D snoRNP biogenesis in response to different growth conditions.

## Results

### R2TP interacts with Nop58 through Pih1

**Figure 1:**
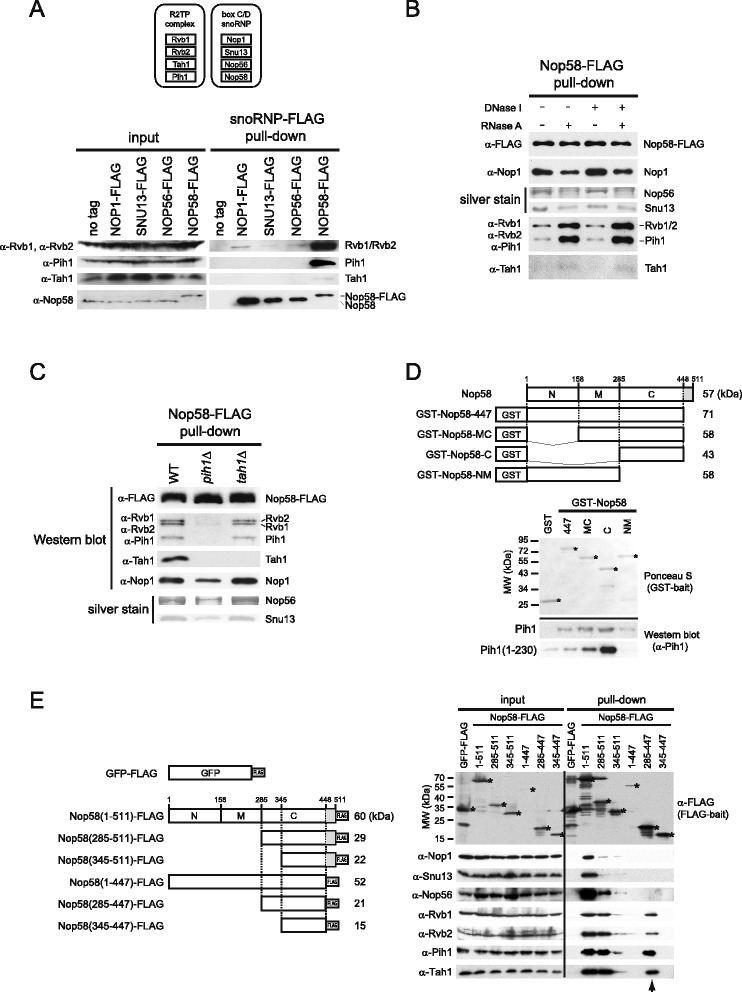
**Interaction between the R2TP complex and Nop58. (A)** Protein components of the R2TP and box C/D snoRNP complexes are shown on top. On bottom are the Western blot analyses for the presence of R2TP proteins in FLAG-tagged Nop1, Snu13, Nop56, and Nop58 pulldown complexes from log phase cells. Cell lysates from same wet weight of log phase cells were used for each pulldown. **(B)** Western blot and silver stain analysis of Nop58-FLAG pulldown complex purified from nuclease-treated or untreated cell lysates. Nop56 and Snu13 were identified by mass spectrometry. **(C)** Western blot analysis of R2TP complex interaction with Nop58-FLAG complex purified from wildtype, *pih1*Δ, or *tah1*Δ background strains. **(D)** Schematic representations of Nop58 constructs generated for the *in vitro* binding assays are shown on top. The KKE/D charged region of Nop58 (residues 448–511) is shown in gray. *In vitro* binding assay for GST-Nop58 and Pih1 is shown on bottom. Ponceau S staining and Western blot analysis of GST pulldown assays using purified GST-Nop58 constructs incubated with Pih1 or Pih1(1–230). Stars indicate the location of the band of the GST-Nop58 construct being tested. **(E)** On the left are schematic representations of the Nop58-FLAG and GFP-FLAG constructs used for the pulldown assays from yeast cells. The KKE/D charged region (residues 448–511) of Nop58 is shown in gray. Western blot analysis of FLAG pulldown assays is shown on the right. Stars indicate the location of the band of Nop58-FLAG or GFP-FLAG constructs corresponding to the schematic on the left panel. The arrowhead shows the lane for Nop58(285–447)-FLAG construct which binds to R2TP but not snoRNP core proteins.

We previously identified genetic interactions between *RVB1*, *RVB2*, and *PIH1* with *NOP58*, and showed that the R2TP complex is required for box C/D snoRNA accumulation and box C/D snoRNP assembly [14]. Also, Gonzales *et al*. [25] showed a physical interaction between Pih1 and Nop58. However, the detailed molecular basis of R2TP complex function in box C/D snoRNP biogenesis remains unknown. To investigate whether R2TP proteins physically interact with box C/D snoRNP complexes, we constructed C-terminal FLAG-tagged Snu13, Nop1, Nop56, and Nop58 strains, and performed pulldown assays using cell lysate extracted from log phase cells followed by Western blot analysis (Figure 1A). All the R2TP proteins were found to significantly interact with Nop58, while Nop1, Snu13, and Nop56 exhibit weak interaction with Rvb1/2 only (Figure 1A). Subsequently, we treated the soluble cell lysate extracted from the Nop58-FLAG strain with RNase A prior to the pulldown in order to disassemble the snoRNPs by degrading the associated snoRNAs. The lysate was also treated with DNase I to degrade DNA as a control (Figure 1B). RNase A but not DNase I treatment reduced the levels of Snu13, Nop1, and Nop56 bound to Nop58-FLAG indicating that the snoRNPs were partially disassembled as expected, whereas no such dissociation was observed for snoRNPs isolated from DNase I-treated cell lysate. Intriguingly, RNase A treatment resulted in increased interaction of R2TP with Nop58-FLAG compared to no treatment or to DNase I-treated sample. These results suggest that the R2TP complex predominantly interacts with Nop58 that is likely released from box C/D snoRNP complexes and that snoRNAs are not required for the R2TP-Nop58 interaction.

In order to determine which protein component of the R2TP complex mediates the interaction with Nop58, we co-immunopurified Nop58-FLAG complex from WT, *pih1*Δ, and *tah1*Δ log phase cells. The levels of Rvb1, Rvb2, Pih1, Tah1, Snu13, Nop1, and Nop56 bound to Nop58-FLAG were determined by Western blot analysis or silver staining. As shown in Figure 1C, the deletion of *PIH1* but not of *TAH1* significantly reduced the interaction of Rvb1/2 with Nop58. Furthermore, the binding of Tah1 to Nop58-FLAG required Pih1 (Figure 1C). The decreased levels of Nop1, Nop56, and Snu13 interacting with Nop58-FLAG in *pih1*Δ cells (Figure 1C) indicates that the snoRNP complex is not properly assembled in these cells. This is consistent with our previous observations in which we noted that the levels of Nop1 and Snu13 that co-purified with Nop56-FLAG were decreased in *pih1*Δ strain [14]. This result indicates that Pih1 enhances the interaction between R2TP and Nop58.

To determine the binding site of Pih1 on Nop58 (511 amino acids), we generated N-terminal GST fusion constructs of different domains of Nop58 based on the available crystal structures of the equivalent archaeal protein [33,34] and expressed them in *E. coli*. Nop58 can be divided into three functional domains (Figure 1D, top panel): N-terminal domain that binds Nop1, middle domain that includes a coiled-coil motif which mediates self-dimerization, and a C-terminal domain, also known as the Nop domain, that interacts with both L7Ae (Snu13 in yeast) and the box C/D sRNA in archaea. The C-terminal domain is followed by a KKE/D repeat (Figure 1D, top panel). GST-fused full-length Nop58 did not express well in *E. coli* (data not shown); but a construct deleted of the C-terminal KKE/D repeat (Figure 1D, top panel), GST-Nop58-447, expressed well and was purified. Deletion constructs lacking the N-terminal, N-terminal and middle, and C-terminal domains of GST-Nop58-447 were also generated and purified (Figure 1D, bottom panel). Since full-length purified Pih1 (344 amino acids) is unstable and readily aggregates, we also tested N-terminal domain of Pih1, Pih1(1–230), which is more stable and soluble [14,26,35]. Both full-length Pih1, as well as, the N-terminal domain of Pih1(1–230) had the highest affinity for the C-terminal domain of Nop58, GST-Nop58-C, while weaker binding was detected with the other Nop58 constructs (Figure 1D).

To further analyze the interactions among R2TP, Nop58, and the other snoRNP factors *in vivo*, we generated FLAG-tagged Nop58 subdomain constructs in p416GAL vector for the overexpression of the constructs in yeast under *GAL1* promoter. As shown in Figure 1E (left panel), we constructed six FLAG-tagged Nop58 constructs, as well as, GFP-FLAG construct as a negative control. All FLAG-fused proteins were overexpressed after galactose induction, except for Nop58(1–447)-FLAG, and each FLAG-tagged complex was co-immunopurified by using anti-FLAG beads (Figure 1E, right panel). The FLAG-tagged proteins that contain the KKE/D repeat, Nop58(1–511), Nop58(285–511), and Nop58(345–511), migrated slower than their actual molecular weight, which could be due to the relatively high content of positively charged lysine residues in their sequence. After the FLAG pulldown, the levels of interacting R2TP and box C/D snoRNP core proteins were compared by Western blot analysis. All R2TP and snoRNP proteins bound to full-length Nop58(1–511), however, snoRNP proteins bound to Nop58 C-terminal domain (285–511) to a lesser extent, and both snoRNP and R2TP proteins did not interact significantly with Nop58(345–511). Intriguingly, R2TP remained associated with Nop58 C-terminal domain deleted of KKE/D (285–447) whereas the snoRNP proteins did not bind this construct. By comparing the construct of Nop58(285–511) with Nop58(285–447), the difference between the two is the presence or absence of KKE/D repeat, suggesting that the KKE/D region might contribute to the interaction between Nop58 and the other snoRNP core components (see Discussion). No binding was observed between Nop58(345–447) and R2TP or snoRNPs. This suggests that a main R2TP binding site in Nop58 is between residues 285–345 at the C-terminus of Nop58. Hence, in this analysis, we identified the Nop58(285–447) region as an exclusive binding site for the R2TP complex and not for snoRNP core proteins. This again strongly suggests that R2TP associates with unassembled Nop58 *in vivo*.

In summary, the data from Figures 1A-E, indicate that the interaction between R2TP and Nop58 is primarily mediated by the binding of the N-terminal domain of Pih1 to the C-terminal Nop domain of Nop58 and that the association does not require other snoRNP core proteins.

### The R2TP complex stabilizes Nop58

**Figure 2:**
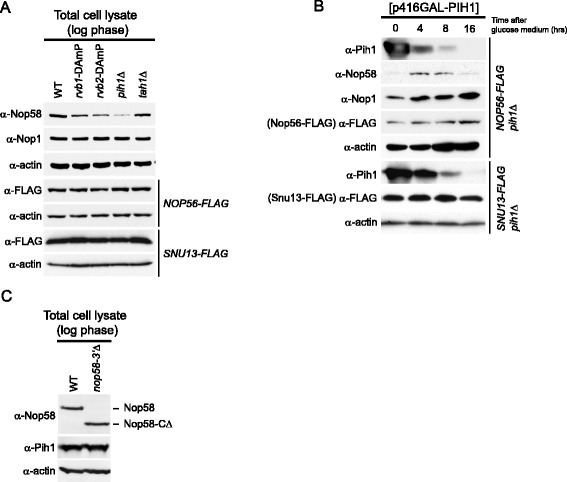
**Effect of R2TP complex on Nop58 stability. (A)** Western blot analysis of steady-state protein levels of box C/D snoRNP protein components in WT, *rvb1*-DAmP, *rvb2*-DAmP, *pih1*Δ, and *tah1*Δ strains in log phase cells. **(B)** Western blot analysis of protein levels of box C/D snoRNP core proteins in *NOP56-FLAG pih1*Δ and *SNU13-FLAG pih1*Δ strains transformed with p416GAL-PIH1 plasmid. Cells were shifted from galactose- to glucose-containing medium, and protein levels were analyzed as a function of time. **(C)** Western blot analysis of Nop58 and Pih1 steady-state levels in log phase WT and *nop58*-3′Δ cells.

To investigate the effect of the R2TP complex on the stability of the core box C/D snoRNP proteins, we assessed the steady-state levels of Nop58, Nop56 (FLAG-tagged), Nop1, and Snu13 (FLAG-tagged) in WT, *rvb1*-DAmP, *rvb2*-DAmP, *pih1*Δ, and *tah1*Δ log phase cells by Western blot analysis. The DAmP strains are hypomorphic alleles of *RVB1* and *RVB2* resulting in lower protein levels than in wildtype cells [36]. Intriguingly, only Nop58 protein levels were decreased in *rvb1*-DAmP, *rvb2*-DAmP, and more considerably in *pih1*Δ. Deletion of *TAH1* had only a slight effect on Nop58 levels. Nop56, Nop1, and Snu13 protein levels were unaffected by the deletion or depletion of the other R2TP proteins (Figure 2A).

We further analyzed the Pih1-dependent stabilization of Nop58 using a conditional expression plasmid p416GAL-PIH1 in which Pih1 expression is under a galactose-inducible promoter. The plasmid was transformed into *NOP56*-FLAG *pih1*Δ cells and *SNU13*-FLAG *pih1*Δ cells. Pre-cultures were grown in galactose minimal media plus raffinose for 16 h until stationary phase to overexpress Pih1, and then inoculated in glucose media to repress the expression of Pih1 from the plasmid. Since Nop1, Nop56, and Nop58 levels are significantly decreased in stationary phase (see below), we maintained the cells in log phase (OD_600_ between 0.3 and 0.6) by continuously adding fresh glucose medium to the culture approximately every 2 h. After the glucose shift, Pih1 levels were drastically reduced over time. In contrast, Nop1, Nop56-FLAG, and Nop58 levels initially increased until 4 h due to growth phase change from stationary to log phase (Figure 2B). After 4 h, Nop1, Snu13-FLAG, and Nop56-FLAG levels remained constant over the time of the experiment, however, Nop58 levels gradually decreased as observed at 8 and 16 h after the shift (Figure 2B). This indicates that Pih1 is required for the stability of Nop58 *in vivo*, but has no effect on the stability of Snu13, Nop1, or Nop56.

We also examined if Nop58 may regulate Pih1 stability by comparing Pih1 protein levels in WT and *nop58-3*′Δ strains. The latter strain, which shows a significant growth defect (data not shown and [14]), expresses a Nop58 mutant missing the last 83 amino acids (Nop58-CΔ) that include part of the Nop domain. However, Pih1 levels were not affected by the functionally deficient Nop58 (Figure 2C).

### The R2TP complex does not interact with box C/D snoRNA

**Figure 3:**
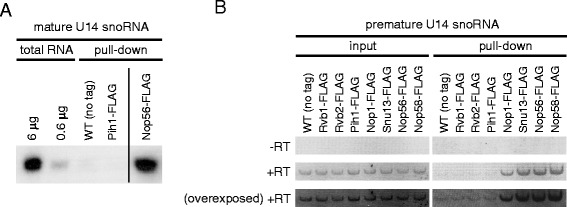
**Analysis of R2TP complex interaction with mature and premature box C/D snoRNA. (A)** Northern blot analysis of mature U14 snoRNA in pulldown complexes of Pih1-FLAG (R2TP) and Nop56-FLAG (box C/D snoRNP); 6 μg and 0.6 μg of total RNA and RNAs purified from the pulldown complexes are shown. WT (no tag) strain was used as a negative control. **(B)** RT-PCR analysis of premature U14 snoRNA in pulldown complexes of R2TP (Rvb1-, Rvb2-, and Pih1-FLAG) and box C/D snoRNP (Nop1-, Snu13-, Nop56-, and Nop58-FLAG). RNAs were purified from the pulldown complexes and PCR was performed with (+RT) or without (−RT) reverse transcription.

To investigate whether R2TP interacts with mature box C/D snoRNAs, we pulled down R2TP or box C/D snoRNP proteins (as a positive control) by performing Pih1-FLAG or Nop56-FLAG pulldowns, respectively. The presence of bound U14 snoRNA was detected by Northern blot analysis. Mature U14 snoRNA co-immunoprecipitated with Nop56-FLAG but not with Pih1-FLAG (Figure 3A).

To investigate the interaction between the R2TP complex and pre-snoRNA, we purified RNA from FLAG pulldown complexes of Rvb1, Rvb2, Pih1, and each of the box C/D snoRNP proteins (Nop1, Snu13, Nop56, and Nop58), and then performed RT-PCR using U14 pre-snoRNA specific primers. All box C/D snoRNP components associated with U14 pre-snoRNA, however, we were not able to detect the pre-snoRNA in Rvb1, Rvb2, and Pih1-FLAG pulldown complexes (Figure 3B). These experiments suggest that the R2TP complex might not significantly interact with mature or premature snoRNA and might not be directly involved in snoRNA processing or maturation.

### The interaction of R2TP with Nop58 is modulated by nucleotide binding

**Figure 4:**
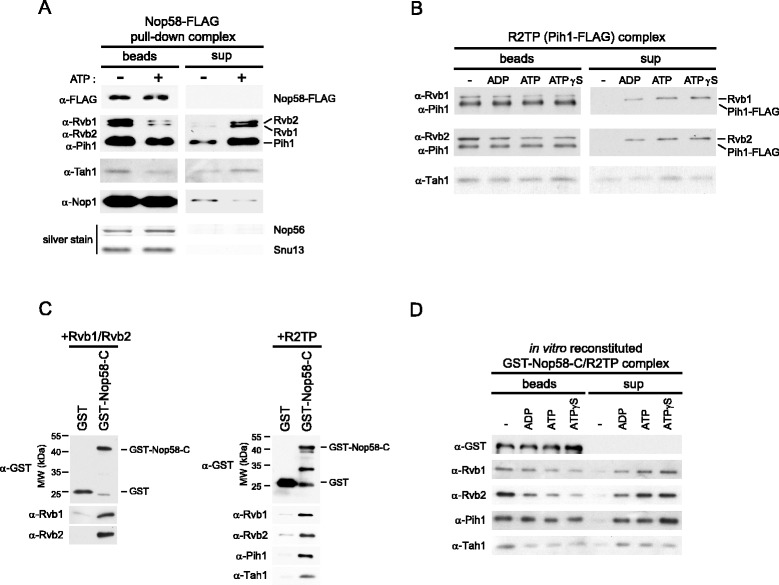
**Effect of nucleotides on the interaction between Nop58 and R2TP. (A)** Western blot or silver stain analysis of Nop58-FLAG pulldown complex in the absence or presence of ATP. The Nop58-FLAG complex purified on α-FLAG beads from cell lysates was incubated with 4 mM ATP for 30 min at 30°C. Supernatant and bead-bound fractions were, subsequently, analyzed. Nop58-FLAG, Rvb1, Rvb2, Pih1, Tah1, and Nop1 were detected by Western blot, while Nop56 and Snu13 were detected by silver stain (and identified by mass spectrometry). **(B)** Western blot analysis of the R2TP (Pih1-FLAG) complex in the absence or presence of different nucleotides. The R2TP complex purified on α-FLAG beads from cells having Pih1 endogenously FLAG-tagged was incubated in the absence or presence of 4 mM ADP, ATP, or ATPγS for 30 min at 30°C. Supernatant and bead-bound fractions were, subsequently, analyzed. **(C)** *In vitro* binding assay for Nop58 C-terminal domain with Rvb1/2 or R2TP complex. GST alone or GST-Nop58-C (residues 285–447) were bound to glutathione-beads and incubated with Rvb1/2 or R2TP complex. After washing the beads, proteins retained on the beads were detected by Western blot analysis. **(D)** Western blot analysis of *in vitro* reconstituted GST-Nop58-C/R2TP complex in the absence or presence of different nucleotides. The GST-Nop58-C/R2TP complex bound to beads prepared as in Figure 4C (right panel) was incubated in the absence or presence of 5 mM ADP, ATP, or ATPγS for 30 min at 30°C. Supernatant and bead-bound fractions were, subsequently, analyzed.

Given that Rvb1 and Rvb2 are AAA + superfamily proteins and have ATPase activity [37] that is known to be essential for snoRNA biogenesis [38], it is very likely that R2TP complex function in box C/D snoRNP assembly is regulated by ATP binding and/or hydrolysis. To investigate the effect of nucleotide on the binding of R2TP to box C/D snoRNP complex, we pulled down the Nop58-FLAG complex from log phase yeast cells using anti-FLAG beads, which also brings down R2TP as shown in Figure 1. The bead-bound complex was then incubated for 30 min at 30°C in the presence or absence of 4 mM ATP. In the presence of ATP, most of snoRNP-bound Rvb1/2 proteins were released into the supernatant; Pih1 and Tah1 were also released but to a lesser extent, while Nop58 and the other box C/D proteins remained largely bound to the beads (Figure 4A). Similar results were obtained using ADP and ATPγS (not shown).

To determine whether the addition of nucleotide to R2TP causes the disassembly of the complex, we immunopurified R2TP from Pih1-FLAG strain using anti-FLAG beads, and then the bead-bound complex was incubated in the absence or presence of 4 mM ADP, ATP, or ATPγS. The levels of the protein components on the beads or in the supernatant fraction were determined by Western blot (Figure 4B). The bait Pih1-FLAG remained tightly retained on the anti-FLAG beads, whereas basal amounts of Tah1 were present in all supernatant fractions, suggesting that the release of Tah1 is not nucleotide dependent and could be a dilution effect. However, Rvb1/2 were dissociated from Pih1-FLAG in the presence of ADP, ATP and ATPγS, indicating that the dissociation of Rvb1/2 from Pih1/Tah1 was induced by nucleotide binding rather than ATP hydrolysis.

Next, we attempted to reconstitute complexes *in vitro*, Nop58 C-terminal domain (residues 285–447) with Rvb1/2 and Nop58 C-terminal domain with R2TP, using purified recombinant proteins from *E. coli*. As shown in Figure 4C, both Rvb1/2 and R2TP formed complexes with glutathione bead-bound GST-Nop58-C, indicating that Rvb1/2 alone is also able to bind the C-terminal domain of Nop58. By using the GST-Nop58-C/R2TP complex, we tested whether nucleotides release the R2TP complex from Nop58 C-terminal domain as observed in Figure 4A. Indeed, R2TP was released from an *in vitro* reconstituted GST-Nop58-C/R2TP complex upon addition of ADP, ATP, and ATPγS (Figure 4D).

Taken together, the results of Figure 4 demonstrate that nucleotide binding to R2TP dissociates the R2TP complex itself and also releases R2TP from Nop58 C-terminal domain.

### The interaction between the R2TP complex and Nop58 is dependent on cell growth phase

**Figure 5:**
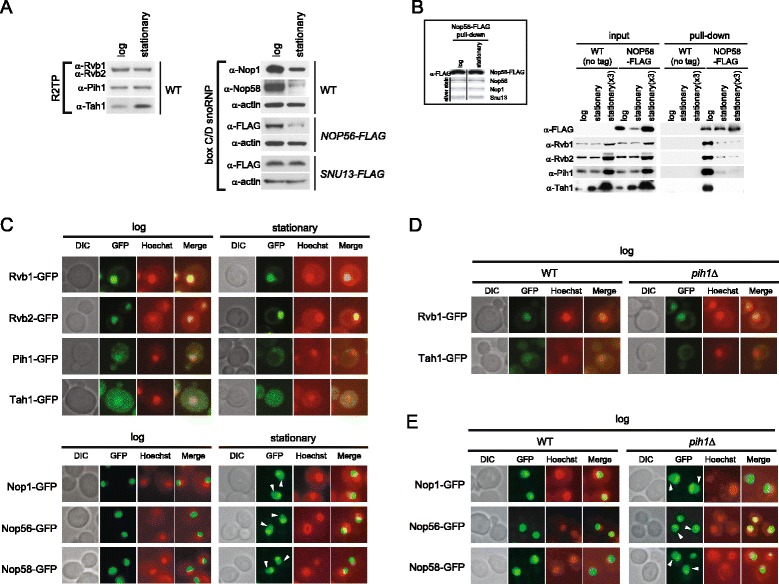
**The dependence of R2TP-Nop58 interaction on cell growth phase. (A)** Western blot analysis of steady-state levels of box C/D snoRNP and R2TP proteins in log and stationary phase cells. **(B)** Western blot analysis of R2TP proteins bound to Nop58-FLAG pulldown complex in log and stationary phase cells. The pulldowns were performed using Nop58-FLAG cell lysates from equal weight of log and stationary phase cell pellets and three times by weight of stationary phase cell pellet [stationary (x3)]. As a negative control, untagged wildtype strain was used. Inset shows the Western blot or silver stain analysis of snoRNP proteins of Nop58-FLAG pulldown purified from log and stationary phase cells. **(C)** Subcellular localizations of endogenously GFP-tagged R2TP and box C/D snoRNP proteins in log and stationary phases. Cells were stained with Hoechst33342 and then analyzed for GFP or Hoechst33342 fluorescence. The images of DIC, GFP (green), and Hoechst33342 (red), and the merged pictures are shown. White arrowheads show nucleoplasmic GFP signals of Nop1-GFP, Nop56-GFP, and Nop58-GFP. **(D)** Subcellular localizations of endogenously GFP-tagged Rvb1 and Tah1 in WT and *pih1*Δ log phase cells. **(E)** Subcellular localizations of endogenously GFP-tagged Nop1, Nop56, and Nop58 in WT and *pih1*Δ log phase cells.

It has been shown that growth phase and nutrient availability actively regulate ribosome biosynthesis including the expression of pre-rRNAs, r-protein genes, and ribosomal biogenesis (Ribi) genes [39]. Given that snoRNP biogenesis is tightly linked with ribosome synthesis, it is reasonable to expect that snoRNP biogenesis is also coordinated with the growth phase of the cell. Therefore, we initially determined the steady-state protein levels of core box C/D snoRNP and R2TP components in log and stationary phase cells. As shown in Figure 5A, Snu13-FLAG, Rvb1, Rvb2, and Pih1 levels did not significantly change between log and stationary phase; however, Nop1, Nop56-FLAG, and Nop58 levels significantly decreased in stationary phase compared to log phase. On the other hand, Tah1 levels were increased in stationary phase relative to log phase (Figure 5A) and this might be related to the role of Tah1 in stress response as an Hsp90 co-factor in addition to its role with the R2TP complex [21].

To test whether the interaction between the R2TP complex and Nop58 is affected by the cell’s growth phase, we performed pulldown assays of Nop58-FLAG from log and stationary phase cells. In this experiment, because of the different levels of the snoRNP proteins between log and stationary phases (Figure 5A), we prepared Nop58-FLAG cell lysates from equal weight (1 g) of log and stationary phase cell pellets but also from three times more by weight (3 g) of stationary phase cells to obtain comparable levels of Nop58-FLAG pulldown complexes. Cell lysates from untagged wildtype cells were also prepared the same way and used as a negative control. As shown in Figure 5B inset, comparable levels of Snu13, Nop1, and Nop56 associated with Nop58 in both log and stationary phases. The interaction of R2TP with Nop58-FLAG was evident in log phase but was significantly reduced in stationary phase and stationary phase (x3) (Figure 5B), suggesting that the interaction between the R2TP complex and Nop58 is controlled by cell growth phase or that there is less free pool of Nop58 in stationary phase cells.

**Figure 6:**
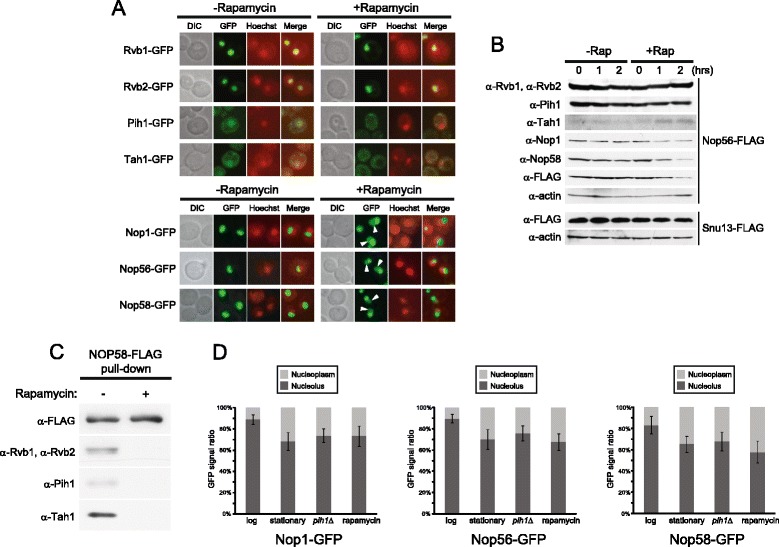
**Effect of different nutrient stresses on the subcellular localization of R2TP and box C/D snoRNP proteins. (A)** Subcellular localizations of endogenously GFP-tagged R2TP and box C/D proteins in log phase cells grown in SD medium in the absence or presence of rapamycin. Cells were stained with Hoechst33342 and then analyzed for GFP or Hoechst33342 fluorescence. The images of DIC, GFP (green), and Hoechst33342 (red), and the merged pictures are shown. White arrowheads show nucleoplasmic GFP signals of Nop1-GFP, Nop56-GFP, and Nop58-GFP. **(B)** Western blot analysis of R2TP and box C/D snoRNP proteins in log phase cells treated or untreated with rapamycin. **(C)** Western blot of Nop58-FLAG pulldown complex in log phase cells in the absence or presence of rapamycin. **(D)** Quantification of Nop1-GFP, Nop56-GFP, and Nop58-GFP signals from nucleoli and nucleoplasms. GFP signals of approximately 100 cells of each strain were quantified using NIH ImageJ.

To determine how the interaction between R2TP and box C/D snoRNP is reduced in stationary phase, we next examined the subcellular localization of the protein components of the R2TP and box C/D snoRNP complexes using endogenously C-terminally GFP-tagged strains. Interestingly, all subunits analyzed in this experiment showed significant subcellular localization changes between log and stationary phases (Figure 5C and Additional file 1: Figure S1). Rvb1-GFP and Rvb2-GFP mainly localized in the nucleus and to a lesser extent to the cytoplasm during log phase as well as stationary phase. Furthermore, we noticed that Rvb1-GFP and Rvb2-GFP form cytoplasmic foci in stationary phase of some cells (Additional file 1: Figure S1). Intriguingly, both Pih1-GFP and Tah1-GFP localized in the nucleus and cytoplasm in log phase, and distributed to the cytoplasm in stationary phase (Figure 5C and Additional file 1: Figure S1). Nop1-GFP, Nop56-GFP, and Nop58-GFP showed dense localization in the nucleolus in log phase, however, the GFP signals diffused into the nucleoplasm in stationary phase. Quantification of the fluorescence signals, showed that 10% to 17% of the box C/D snoRNP proteins can be detected in the nucleoplasm in log phase and that the number increases to 30% to 35% in stationary phase (refer to Figure 6D). This indicates that there is a defect or reduction in the rate of translocation of the snoRNP proteins from the nucleoplasm to the nucleolus in stationary phase. Note that we could not detect a reliable signal for Snu13-GFP. Hence, the subcellular localizations of the R2TP and snoRNP components dynamically changes based on growth phase, which could explain the growth phase-dependent interaction between R2TP and Nop58 (Figure 5B).

### Pih1 is required for the proper localization of R2TP and box C/D snoRNP proteins

In previous studies, it was shown that Rvb2 depletion as well as *PIH1* deletion caused delocalization of snoRNP proteins [25,38]. Since Pih1 seems to act as an adaptor between R2TP and Nop58 (Figure 1C,E), we examined how Pih1 might affect R2TP and snoRNP localizations by observing GFP fusion proteins of all related components in *pih1*Δ background. In log phase *pih1*Δ cells, less Tah1-GFP was present in the nucleus compared to WT cells (Figure 5D and Additional file 2: Figure S2). On the other hand, there was no significant change in Rvb1-GFP localization between WT and *pih1*Δ cells (Figure 5D and Additional file 2: Figure S2). This could reflect the fact that Rvb1/2 are involved in many other nuclear complexes in addition to R2TP [24] and that the levels of Rvb1/2 in the cell are much higher than that of Pih1 (unpublished data). Interestingly, the deletion of *PIH1* led to increased nucleoplasmic localization of Nop1, Nop56, and Nop58 (Figure 5E, Figure 6D, and Additional file 3: Figure S3), which is consistent with the proposed role of Pih1 in the nucleolar localization of box C/D core proteins [25]. These results suggest that Pih1 maintains the nuclear localization of R2TP and is required for the proper localization of the box C/D snoRNPs in the nucleolus.

### Nutrient starvation affects the subcellular localization of R2TP and box C/D snoRNP proteins

The above localization results led us to investigate the subcellular distribution of the R2TP and box C/D snoRNP proteins in response to different nutrient starvation conditions such as carbon starvation, nitrogen starvation, and rapamycin treatment. Rapamycin inactivates the TOR nutrient signaling pathway and induces a starvation-like state in yeast [40]. As in stationary phase cells (Figure 5C), Pih1 and Tah1 delocalized to the cytoplasm from the nucleus upon rapamycin treatment, while Nop1, Nop56, and Nop58 diffused into the nucleoplasm from the nucleolus (Figure 6A,D). Similar relocalization patterns were observed under carbon and nitrogen starvation conditions (Additional file 4: Figure S4). Rvb1 and Rvb2 showed no drastic changes in their localizations under these conditions consistent with the results of Figure 5C.

Levels of all box C/D snoRNP proteins, except for Snu13, decreased upon rapamycin treatment (Figure 6B), as seen in stationary phase cells (Figure 5A). Also, the interaction between R2TP and Nop58-FLAG significantly decreased in rapamycin-treated cells (Figure 6C). The results of Figures 5 and 6 suggest that nutrient starvation destabilizes the box C/D snoRNP complex as a result of the dynamic nucleo-cytoplasmic translocation of the R2TP complex, which could consequently reduce the levels of box C/D snoRNPs translocated or the rate of translocation from the nucleoplasm to the nucleolus.

### Subcellular localization of the R2TP complex is dependent on nucleo-cytoplasmic transport system

**Figure 7:**
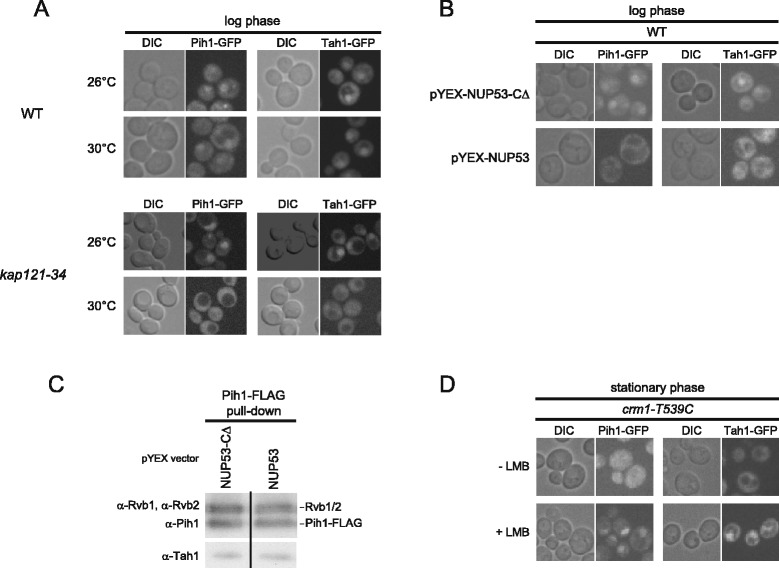
**R2TP nuclear import and export is dependent on Kap121 and Crm1. (A)** Subcellular localizations of endogenously GFP-tagged Pih1 and Tah1 in wildtype background and in *kap121-34* mutant cells at the permissive (26°C) and restrictive (30°C) temperatures in log phase cells. **(B)** Subcellular localizations of endogenously GFP-tagged Pih1 and Tah1 in WT cells expressing Nup53-CΔ or Nup53 under *CUP1* promoter in log phase cells. **(C)** Western blot analysis of R2TP complex purified from log phase cells expressing Nup53-CΔ or Nup53. **(D)** Subcellular localizations of endogenously GFP-tagged Pih1 and Tah1 in *crm1*-T539C background strain in the absence or presence of LMB in stationary phase.

We asked whether the dynamic translocation of R2TP complex in different growth phases and nutrient conditions is dependent on the nucleo-cytoplasmic transport system. First, we examined the dependence of Pih1 and Tah1 nuclear import on Kap121, which is one of the major karyopherins involved in nuclear import [41]. The localization of endogenously C-terminally GFP-tagged Pih1 and Tah1 were analyzed in wildtype and *kap121-34* temperature sensitive mutant allele at the permissive (26°C) and restrictive temperatures (30°C). Cells were grown to early-log phase at the permissive temperature and further incubated for 2 h at 26°C or 30°C. In wildtype cells, Pih1-GFP and Tah1-GFP localized mainly in the nucleus with some distribution of GFP-tagged proteins in the cytoplasm at both 26°C and 30°C (Figure 7A). In *kap121-34* mutant, similar localizations of Pih1-GFP and Tah1-GFP were observed at 26°C as in wildtype cells, however, both Pih1-GFP and Tah1-GFP delocalized to the cytoplasm upon inactivation of Kap121 at 30°C (Figure 7A). Hence, Kap121 mediates the nuclear import of Tah1 and Pih1. To further confirm this result, a parallel experiment was carried out using cells overexpressing either Nup53, which is known to significantly inhibit the Kap121-mediated nuclear import pathway, or Nup53-CΔ, which cannot inhibit Kap121 activity since it lacks the Kap121 binding domain (residues 405–430) [42]. Consistent with the above results, overexpression of Nup53 greatly inhibited the nuclear import of both Pih1-GFP and Tah1-GFP, whereas the overexpression of Nup53-CΔ did not inhibit the translocation (Figure 7B), showing that the nuclear import of Pih1 and Tah1 is Kap121-dependent. We did not observe a significant change in the subcellular localization of Rvb1-GFP and Rvb2-GFP in Nup53-CΔ *vs*. Nup53 overexpressing cells (Additional file 5: Figure S5). This might reflect the fact that a large amount of Rvb1 and Rvb2 are part of other complexes such as Ino80 and SWR-C chromatin remodeling complexes in the nucleus and, hence, their nuclear import might be independent of Kap121 activity. However, pulldown of the R2TP complex using Pih1-FLAG from Nup53-CΔ and Nup53 overexpressing cells at log phase showed no changes in the stoichiometry of the complex (Figure 7C) suggesting that all the R2TP protein components tightly form a complex and translocate together between the nucleus and cytoplasm.

Next, we analyzed the dependence of R2TP complex export on Crm1, which is one of the main exportins of ribosomes in eukaryotes [43,44]. To inhibit the activity of Crm1, we used cells expressing *crm1-T539C* mutant, which is sensitive to leptomycin B (LMB), an inhibitor of Crm1 [45]. In these cells, Pih1-GFP and Tah1-GFP delocalized to the cytoplasm from the nucleus in stationary phase cells in the absence of LMB (Figure 7D) as seen in Figure 5C, however, the two proteins remained in the nucleus in the presence of LMB (Figure 7D). Thus, our observations suggest that the dynamic nucleo-cytoplasmic translocation of the R2TP complex in different growth phases is dependent on the karyopherins, Kap121, and Crm1. Note that we were not able to detect direct interactions between Kap121 or Crm1 and R2TP (data not shown), which suggests that R2TP localization is indirectly regulated by the respective importin and exportin or that the interactions are weak.

### Nuclear localization of R2TP is important for proper cell growth

**Figure 8:**
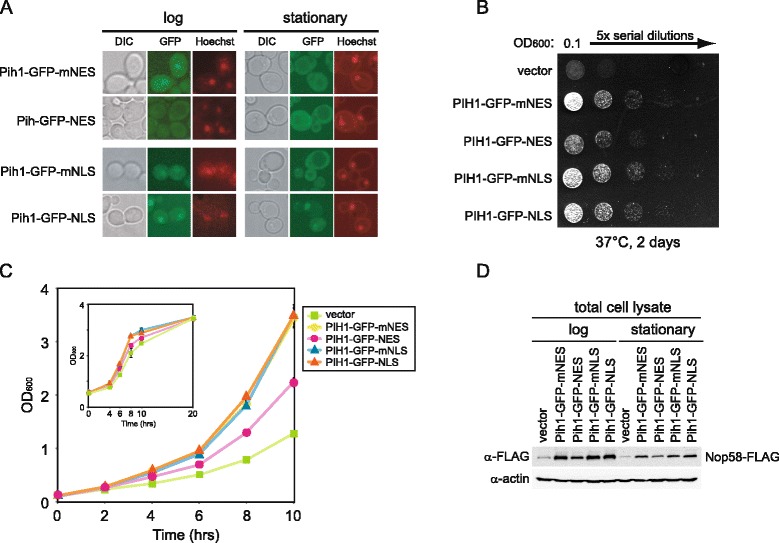
**Effect of targeting Pih1 to cytoplasm or nucleus on cell growth. (A)** Pih1-GFP was fused with nuclear export signal (NES), mutagenized nuclear export signal (mNES), nuclear localization signal (NLS), or mutagenized nuclear localization signal (mNLS), and expressed from pRS315 vector under the *PIH1* promoter in Nop58-FLAG *pih1*Δ strain. Cells were stained with Hoechst33342 and then analyzed for GFP or Hoechst33342 fluorescence. The images of DIC, GFP (green), and Hoechst33342 (red) are shown. **(B)** Dilution spot assay of the strains described in A. Cells were serially diluted fivefold, spotted onto SD-Leu plate, and incubated for 2 days at 37°C. **(C)** Growth curves in liquid culture for the different indicated strains. Cells were grown in SD-Leu medium at 37°C and OD_600_ was measured every 2 h. Inset shows growth curve of the same strains until stationary phase. **(D)** Western blot analysis of total cell lysate of the different transformants. Cell lysates were prepared from cells grown at 37°C until log or stationary phase.

To investigate whether the nuclear-cytoplasmic shuttling of R2TP has an effect on cell growth, we prevented or reduced R2TP shuttling by adding a nuclear export signal (NES) or a nuclear localization signal (NLS) to the Pih1 sequence. We generated constructs which express Pih1-GFP fused to the NES derived from human protein kinase A inhibitor (LALKLAGLDINKT) or to the NLS from SV40 T antigen (PKKKRKV) [46] controlled by *PIH1* native promoter. As controls, plasmids expressing Pih1-GFP with non-functional mutant NES (LALKLAG**A**D**T**NKT) or NLS (PK**A**KRKV) denoted as mNES and mNLS, respectively, were also used [46]. The constructs were transformed into Nop58-FLAG *pih1*Δ background strain and GFP localization was observed using fluorescence microscopy. Pih1-GFP-NES and Pih1-GFP-NLS remained in the cytoplasm and in the nucleus, respectively, in both log and stationary phases (Figure 8A). On the other hand, Pih1-GFP-mNES and Pih1-GFP-mNLS changed localization between the nucleus in log phase and the cytoplasm in stationary phase (Figure 8A) as observed for untagged Pih1-GFP (Figure 5C).

To analyze the effect of forcing Pih1 localization to the cytoplasm or nucleus, we compared cell growth of different transformants. Intriguingly, whether on plates or in liquid culture, we observed a slower growth phenotype for *pih1*Δ cells expressing Pih1-GFP-NES compared to *pih1*Δ cells expressing Pih1-GFP-NLS, Pih1-GFP-mNES, or Pih1-GFP-mNLS (Figure 8B,C). We did not observe a faster growth or delay in entering stationary phase for the Pih1-GFP-NLS transformant compared to Pih1-GFP-mNLS (Figure 8C inset). Also, Nop58-FLAG protein levels were decreased in both log and stationary phase in *pih1*Δ cells expressing Pih1-GFP-NES (or in cells having vector only) compared to other transformants. It should also be noted that Pih1-GFP-NLS does not restore the levels of Nop58-FLAG present in stationary phase to those observed in log phase (Figure 8D). This suggests that other additional cellular mechanisms control Nop58 levels in stationary phase.

In summary, the results of Figure 8 suggest that the nuclear localization of the R2TP complex is required for its proper function in maintaining Nop58 stability and in controlling box C/D snoRNP biogenesis.

## Discussion

Our data clearly demonstrate that the R2TP complex plays an essential role in stabilizing Nop58 in the process of box C/D snoRNP maturation (Figures 1 and 2). Intriguingly, this R2TP function is influenced by growth phases and nutrient conditions and is regulated by the subcellular localization of its protein components (Figures 5, 6, and 7).

The depletion or deletion of the R2TP components, except for Tah1, results in reduced Nop58 levels (Figure 2A and B). This result suggests that the main functional components of the R2TP complex for snoRNP biogenesis are Rvb1, Rvb2, and Pih1, while Tah1 could be an accessory protein. This is consistent with our previous result showing that Tah1 effect on snoRNP biogenesis is subtle, and that Tah1 functions with Hsp90 to maintain Pih1 protein stability [14]. Although we observed a direct interaction of Rvb1/2 with Nop58 C-terminal domain *in vitro* (Figure 4C, left panel), the Nop58-FLAG pulldown experiment in *pih1*Δ showed a significant reduction of Rvb1/2 binding with Nop58, indicating that the interaction of the R2TP complex with Nop58 is largely mediated by Pih1 *in vivo* (Figure 1C). More specifically, we found that the N-terminal domain of Pih1, residues 1–230, binds to the C-terminal Nop domain of Nop58 (Figure 1D). Furthermore, Pih1(1–230) was also found to interact with Rvb1/2, but the interaction was significantly enhanced for Pih1(1–248) [35]. We consider Pih1 to define the R2TP complex in yeast.

We found that the R2TP complex significantly interacts with the C-terminal domain of Nop58 spanning residues 285–447 (Figure 1E). No binding of snoRNP core proteins to this region of Nop58 was detected suggesting that R2TP interacts with Nop58 that has not been assembled with other snoRNP core factors. Unexpectedly, it was also observed that deletion of the KKE/D region from Nop58 C-terminus (residues 448–511) significantly reduced Nop58 interaction with the other snoRNP core proteins, suggesting that this charged region of Nop58 might contribute to the association between Nop58 and other snoRNP core factors. In yeast, the deletion of KKE/D does not significantly affect cell growth and box C/D snoRNA binding but causes a loss of the compaction of the nucleolus [47-49]. Also, it has been found that this highly charged domain of Nop58 interacts with Tgs1 *in vitro*; Tgs1 is a tri-methylguanosine synthase and is essential for hypermethylation of the 5' m^7^G cap of snRNAs and snoRNAs [50]. Furthermore, in mammalian cells, the C-terminal domain of Nop58 containing the charged region is essential for the nucleolar localization of Nop58 and functions as a nucleolar localization signal (NoLS) [20]. Taken together, these observations suggest an important role for the KKE/D region of Nop58 in snoRNP biogenesis. It would be important to further analyze the physiological role of this domain.

If the yeast and archaeal box C/D complexes have the same general arrangement [33,34], then the Nop domain of Nop58 has multiple binding partners including the snoRNA, Snu13, and Pih1. Furthermore, based on the recent X-ray crystal structure of the archaeal box C/D RNA-protein complex [34], it is clear that Nop58 is a key component of the complex providing an interaction platform for the other core proteins and for the snoRNA.

Recently, it has been reported that Pih1-Tah1 heterodimer interacts with Snu13-U14 snoRNA through Rsa1 *in vitro* [51]. However, we were not able to identify an interaction between Snu13 and Pih1-Tah1 *in vivo* (Figure 1A). We also performed Rsa1-FLAG pulldowns using yeast soluble cell lysate but no interaction with R2TP components was detected (data not shown), suggesting that the Pih1-Tah1-Snu13-snoRNA-Rsa1 interaction might be weak or transient under physiological condition.

Intriguingly, the R2TP complex interacts with the unassembled form of Nop58 with high affinity (Figure 1A,B,E), but does not interact with either mature or premature box C/D snoRNA (Figure 3). These results suggest that R2TP is involved in a very early stage of the box C/D snoRNP biogenesis before Nop58 assembles with the other snoRNP components: Snu13, Nop1, Nop56, and snoRNAs. In a previous study, we showed that the deletion or depletion of R2TP components affects the accumulation of mature box C/D snoRNAs [14]. This phenotype could be the result of the destabilization of Nop58. The interaction between R2TP and Nop58 is evident in log phase cells that require high production rate of snoRNPs for efficient ribosome biogenesis but not in stationary phase cells. Therefore, it is likely that the total box C/D snoRNP levels in the cell, and consequently pre-rRNA processing, could be effectively regulated by modulating Nop58 levels rather than other box C/D snoRNP core components. This would explain why the R2TP complex, by affecting the stability of Nop58, plays a critical role in cell physiology. Previously, we reported that R2TP is involved in snoRNP biogenesis in both log and stationary phases [14]. As mentioned above, we observed a strong interaction between R2TP and Nop58 in log phase whereas it was weaker in stationary phase. However, this low level of binding could be sufficient in stationary phase cells to maintain the basal levels of Nop58 and, subsequently, conserve snoRNA production.

In mammalian cells, it has been reported that the treatment of human cells by proteasome inhibitor MG-132 leads to improper localization of Nop58 [52]. Furthermore, post-translational modifications of mammalian Nop58 have also been reported [53,54]. The protein has been found to be phosphorylated by casein kinase II (CK2), and, subsequently, SUMOylated to increase its stability. These observations further highlight the importance of regulating Nop58 stability. It will be interesting to identify such modifications for yeast Nop58 and to determine how the modifications affect Nop58 stability, function, and Nop58-R2TP interaction in response to different growth and nutrient conditions.

Based on the localization studies (Figures 5C, 6A, and 7A,B,D), we unexpectedly found that the ability of the R2TP complex to modulate the assembly of box C/D snoRNPs is regulated by a nucleo-cytoplasmic shuttling mechanism that is dependent on the Kap121 and Crm1 karyopherins, which are also known to be involved in nucleo-cytoplasmic trafficking of ribosomal proteins [41,43]. The shuttling of the R2TP proteins between the nucleus and the cytoplasm is growth phase and nutrient dependent. In stationary phase cells (Figure 5C) or in the absence of carbon or nitrogen sources (Additional file 4: Figure S4), or in the presence of rapamycin (Figure 6A), we observed that some of the R2TP complex shuttles out of the nucleus, all core box C/D snoRNP protein levels, except for Snu13, are reduced (Figure 5A and 6B) and a larger proportion of the snoRNP proteins are present in the nucleoplasm (Figures 5C, 6A, and Additional file 4: Figure S4). In contrast, when nutrients are available, then the R2TP complex is enriched in the nucleus (Figure 5C), all core box C/D snoRNP protein levels were recovered (Figures 5A and 6B) and localized in the nucleolus (Figure 5C). As shown in Figure 8, this shuttling of the R2TP complex actively influences cell growth. It is known that ribosome synthesis is the major energy consuming process in the cell [2], and that ribosome activity is tightly coupled to growth phase and nutrient availability [39]. Hence, the relocalization of the R2TP complex allows the cells to rapidly respond and control snoRNP biogenesis, and, subsequently, regulate pre-rRNA processing and ribosome biogenesis in response to different growth conditions.

It is interesting to note that Rix7, which is an essential AAA + ATPase required for the biogenesis and nuclear export of 60S ribosomal subunits, also undergoes growth phase redistribution [43]. It localizes throughout the nucleus in exponentially growing cells, but concentrates in the nucleolus in stationary phase cells. Hence, the dynamic relocalization of protein complexes involved directly or indirectly in ribosome biogenesis might be a generally conserved mechanism designed to allow the cell to easily correlate the number of ribosomes to nutrient availability and growth phase.

It is known that ribosome biogenesis is regulated by the TOR signaling pathway [40], which balances the production of ribosome components to nutrient availability. We observed that the R2TP complex is regulated by the TOR signaling pathway since the localization of the R2TP proteins is affected by the specific inhibitor for TOR, rapamycin. At this stage, we do not know how the TOR pathway effects the nucleo-cytoplasmic translocation of the R2TP complex. However, one intriguing finding from our study points to the presence of a signaling-like pathway that links chaperone activity to ribosome biogenesis, which is based on stabilizing protein complexes (Additional file 6: Figure S6). In our earlier study [14], we had shown that the stability of Pih1 depends on the activity of Hsp90 chaperone together with its co-factor Tah1. This activity then results in the proper assembly of the R2TP complex. In this study, we demonstrated that the proper function and localization of the R2TP complex is required for the stability of Nop58 and assembly of the box C/D snoRNP complexes. The critical players in this pathway are Hsp90, Pih1, and Nop58; both Pih1 and Nop58 are unstable proteins. Importantly, there seems to be a directionality to this signaling-like pathway since functionally deficient Nop58 does not affect Pih1 (Figure 2C), while the destabilization or deletion of Pih1 does affect Nop58 (Figure 2A,B). The presence of such signaling-like pathways that are based on the stabilization of protein complexes rather than protein modification might be widespread in the cell and warrants further investigation.

## Conclusions

**Figure 9:**
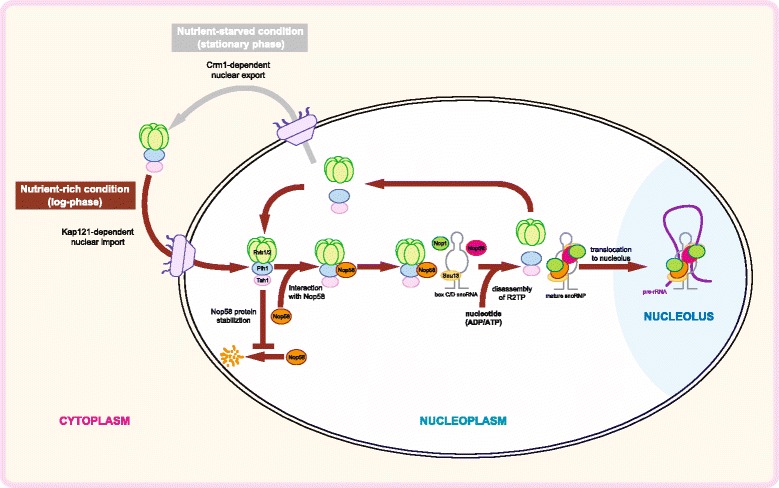
**A model of the regulation of box C/D snoRNP biogenesis by the R2TP complex.** The R2TP complex regulates the stability of unassembled Nop58 and modulates box C/D snoRNP biogenesis by changes in the subcellular distribution of its protein components in response to growth phase and nutrient conditions. The subcellular translocation of R2TP complex is Kap121- and Crm1-dependent.

Figure 9 provides a summary of our current model of the regulation of box C/D snoRNP biogenesis by the R2TP complex. Most of the R2TP complex localizes in the nucleus in log phase or nutrient rich conditions and associates with the free pool of Nop58 that has not yet assembled with other box C/D snoRNP components. The R2TP-Nop58 interaction stabilizes Nop58, prevents its degradation, and eventually promotes its assembly into the box C/D snoRNP complex. We observed that the R2TP complex dissociates from box C/D snoRNPs and that the complex itself disassembles in the presence of nucleotides (Figure 4). Hence, upon nucleotide binding by R2TP, the mature snoRNP then translocates to the nucleolus, while R2TP is recycled. In exponentially growing cells, the R2TP complex is enriched in the nucleus by a Kap121-dependent mechanism to function in the assembly of box C/D snoRNPs. However, in stationary phase or under nutrient limiting conditions, the R2TP complex is actively translocated to the cytoplasm by a Crm1-dependent pathway, and as a result, the assembly of the box C/D snoRNPs is affected, which eventually results in the decrease of ribosome biogenesis. Hence, the regulated subcellular relocalization of R2TP modulates ribosome biogenesis.

## Materials and methods

### Yeast strains and media

*S. cerevisiae* strains used in this work are listed in Table 1. C-terminal 3xFLAG-tagged strains and gene deletion mutants were constructed by PCR-based homologous recombination method [55]. Standard media, YPD, YPGal, and SD containing appropriate supplements were made as described previously [56]. Unless stated otherwise, yeast strains were grown at 30°C. For rapamycin treatment, log phase cells (OD_600_ = 0.3) in SD medium were treated with 100 ng/mL rapamycin for 2 h. For carbon or nitrogen starvation, cells were grown to log phase (OD_600_ = 0.3) in SD medium, and washed with distilled water, then incubated in carbon-depleted medium (SD without 2% dextrose) or nitrogen-depleted medium (0.17% yeast nitrogen base without amino acids and ammonium sulfate, and 2% glucose) for 2 h. For leptomycin B treatment, log phase cells (OD_600_ = 0.3) were incubated with 20 ng/mL of leptomycin B for 16 h until stationary phase.

**Table 1:** **Yeast strains used in this study**

**Name**	**Background**	**Genotype**	**References**
Y239	S288C	*MATa his3Δ1 leu2Δ0 met15Δ0 ura3Δ0*	
Y240	S288C	*MATalpha his3Δ1 leu2Δ0 met15Δ0 ura3Δ0*	
YK32	S288C	*MATa his3Δ1 leu2Δ0 met15Δ0 ura3Δ0 NOP1-FLAG::KANMX*	This study
YK68	S288C	*MATalpha his3Δ1 leu2Δ0 met15Δ0 ura3Δ0 SNU13-FLAG::KANMX*	This study
YK3	S288C	*MATa leu2 his3 ura3 lys2 NOP56-FLAG::KANMX*	[14]
YK23	S288C	*MATalpha his3Δ1 leu2Δ0 met15Δ0 ura3Δ0 NOP58-FLAG::KANMX*	This study
YK28	S288C	*MATalpha his3Δ1 leu2Δ0 met15Δ0 ura3Δ0 NOP58-FLAG::KANMX pih1Δ::URA3*	This study
YK29	S288C	*MATalpha his3Δ1 leu2Δ0 met15Δ0 ura3Δ0 NOP58-FLAG::KAN tah1Δ::URA3*	This study
YK26	S288C	*MATalpha his3Δ1 leu2Δ0 met15Δ0 ura3Δ0 RVB1-FLAG::KANMX*	This study
YK27	S288C	*MATalpha his3Δ1 leu2Δ0 met15Δ0 ura3Δ0 RVB2-FLAG::KANMX*	This study
YK31	S288C	*MATa his3Δ1 leu2Δ0 met15Δ0 ura3Δ0 PIH1-FLAG::KANMX*	This study
*rvb1*-DAmP	S288C	*MATalpha can1Δ ::STE2pr-Sp_his5 lyp1Δ ::STE3pr-LEU2 RVB1-3'UTR::NAT*	[36]
*rvb2*-DAmP	S288C	*MATalpha can1Δ ::STE2pr-Sp_his5 lyp1Δ ::STE3pr-LEU2 RVB2-3'UTR::NAT*	[36]
R0055	S288C	*MATalpha can1Δ∷MFA1pr-HIS3 his3Δ1 leu2Δ0 ura3Δ0 met15Δ0 lys2Δ0 pih1Δ∷NAT*	[21]
R0061	S288C	*MATalpha can1Δ∷STE2pr-Sp_his5 lyp1Δ his3Δ1 leu2Δ0 ura3Δ0 met15Δ0 tah1Δ∷URA3*	[14]
*nop58-3′Δ*	S288C	*MATa his3Δ1 leu2Δ0 met15Δ0 ura3Δ0 yor309cΔ∷KANMX*	Open Biosystems
RVB1-GFP	S288C	*MATa his3Δ1 leu2Δ0 met15Δ0 ura3Δ0 RVB1-GFP::HIS3*	Life Technologies
YK169	S288C	*MATa his3Δ1 leu2Δ0 met15Δ0 ura3Δ0 RVB2-GFP::KANMX*	This study
PIH1-GFP	S288C	*MATa his3Δ1 leu2Δ0 met15Δ0 ura3Δ0 PIH1-GFP::HIS3*	Life Technologies
TAH1-GFP	S288C	*MATa his3Δ1 leu2Δ0 met15Δ0 ura3Δ0 TAH1-GFP::HIS3*	Life Technologies
NOP1-GFP	S288C	*MATa his3Δ1 leu2Δ0 met15Δ0 ura3Δ0 NOP1-GFP::KANMX*	This study
NOP56-GFP	S288C	*MATa his3Δ1 leu2Δ0 met15Δ0 ura3Δ0 NOP56-GFP::HIS3*	Life Technologies
NOP58-GFP	S288C	*MATa his3Δ1 leu2Δ0 met15Δ0 ura3Δ0 NOP58-GFP::HIS3*	Life Technologies
*RVB1-GFP pih1Δ*	S288C	*MATa his3Δ1 leu2Δ0 met15Δ0 ura3Δ0 RVB1-GFP::HIS3 pih1Δ::NAT*	This study
*TAH1-GFP pih1Δ*	S288C	*MATa his3Δ1 leu2Δ0 met15Δ0 ura3Δ0 TAH1-GFP::HIS3 pih1Δ::NAT*	This study
*NOP1-GFP pih1Δ*	S288C	*MATa his3Δ1 leu2Δ0 met15Δ0 ura3Δ0 NOP1-GFP::KANMX pih1Δ::NAT*	This study
*NOP56-GFP pih1Δ*	S288C	*MATa his3Δ1 leu2Δ0 met15Δ0 ura3Δ0 NOP56-GFP::HIS3 pih1Δ::NAT*	This study
*NOP58-GFP pih1Δ*	S288C	*MATa his3Δ1 leu2Δ0 met15Δ0 ura3Δ0 NOP58-GFP::HIS3 pih1Δ::NAT*	This study
*kap121-34* PIH1-GFP	DF5	*MATa ura3-52 his3-Δ200 trp1-1 leu2-3,112 lys2-801 kap121Δ::LEU2 pkap121-34-TRP1 PIH1-GFP::HIS3*	This study
*kap121-34* TAH1-GFP	DF5	*MATa ura3-52 his3-Δ200 trp1-1 leu2-3,112 lys2-801 kap121Δ::LEU2 pkap121-34-TRP1 TAH1-GFP::HIS3*	This study
YK124	S288C	*MATa his3Δ1 leu2Δ0 met15Δ0 ura3Δ0 PIH1-GFP::HIS3* [pYEX-NUP53]	This study
YK125	S288C	*MATa his3Δ1 leu2Δ0 met15Δ0 ura3Δ0* [pYEX-NUP53 *Δ*405-430]	This study
YK175	S288C	*MATa his3Δ1 leu2Δ0 ura3Δ0 crm1-T539C PIH1-GFP::HIS3*	This study
YK176	S288C	*MATa his3Δ1 leu2Δ0 ura3Δ0 met15Δ0 crm1-T539C TAH1-GFP::HIS3*	This study

### Plasmids

For expression of GST-tagged Nop58 variants in *E. coli*, four constructs were prepared, pGST-Nop58-447, pGST-Nop58-MC, pGST-Nop58-C, and pGST-Nop58-NM (Figure 1D). The yeast *NOP58* gene was amplified by PCR from genomic DNA using the primers listed in Table 2 and then cloned into *Bam*HI-*Xho*I sites of pGEX-6P-1 vector (GE Healthcare). For overexpression of Nop58-FLAG subdomains for pulldown experiments in yeast, seven constructs were prepared including a negative control, p416GAL-Nop58(1–511)-FLAG, p416GAL-Nop58(285–511)-FLAG, p416GAL-Nop58(345–511)-FLAG, p416GAL-Nop58(1–447)-FLAG, p416GAL-Nop58(285–447)-FLAG, p416GAL-Nop58(345–447)-FLAG, and p416GAL-GFP-FLAG. For p416GAL-Nop58(1–511)-FLAG, p416GAL-Nop58(285–511)-FLAG, and p416GAL-Nop58(345–511)-FLAG, the FLAG-tagged *NOP58* DNA fragments were amplified from genomic DNA of NOP58-FLAG strain by PCR using primers listed in Table 2 and inserted into *Bam*HI-*Xho*I sites of p416GAL. For p416GAL-Nop58(1–447)-FLAG, p416GAL-Nop58(285–447)-FLAG, and p416GAL-Nop58(345–447)-FLAG constructs, the *NOP58* DNA fragments were amplified from wildtype yeast genomic DNA using primers listed in Table 2. The GFP DNA sequence was amplified by PCR from pFA6a-GFP(S65T)-KanMX plasmid using primers in Table 2 and cloned into *Bam*HI-*Xho*I sites of p416GAL vector.

**Table 2:** **Primers used in this study for PCR**

**Plasmid/Gene names**	**Primers**	**Sequences**
pGEX-Nop58-447	NOP58-BHI-F	TTGGATCCATGGCTTACGTTTTAACTGAAACTTC
	NOP58-447-XhoI-R	TTTCTCGAGTTATTCATCATCAGAATCGGATTCAGAATC
pGEX-Nop58-MC	NOP58-472-BamHI-F	TTGGATCCATGAAAGTTGATGTTATGATTATTCAAGCA
	NOP58-447-XhoI-R	TTTCTCGAGTTATTCATCATCAGAATCGGATTCAGAATC
pGEX-Nop58-C	NOP58-853-BamHI-F	TTGGATCCATGCCAAACTTGACTCAGTTGGTTG
	NOP58-447-XhoI-R	TTTCTCGAGTTATTCATCATCAGAATCGGATTCAGAATC
pGEX-Nop58-NM	NOP58-BHI-F	TTGGATCCATGGCTTACGTTTTAACTGAAACTTC
	NOP58-852-XhoI-R	TTTCTCGAGTTAAGCAATAGCCTTCATTCTTGCAG
p416GAL-GFP-FLAG	GFPs65t-BamHI-F	TTGGATCCATGAGTAAAGGAGAAGAACTTTTCACTGGAG
	GFP-nonstop-XhoI-R	TTTCTCGAGTTTGTATAGTTCATCCATGCCATG
p416GAL-Nop58(1–511)-FLAG	NOP58-BHI-F	TTGGATCCATGGCTTACGTTTTAACTGAAACTTC
	FLAG-XhoI-R	TTTCTCGAGCTATTTATCGTCATCATCTTTGTAGTCCTTGTC
p416GAL-Nop58(285–511)-FLAG	NOP58-853-BamHI-F2	TTGGATCCATGCCAAACTTGACTCAGTTGGTTGGTGAAT
	FLAG-XhoI-R	TTTCTCGAGCTATTTATCGTCATCATCTTTGTAGTCCTTGTC
p416GAL-Nop58(345–511)-FLAG	NOP58-1033-BamHI-F	TTGGATCCATGGCCTCTCTTGTTGGTCAAGCTACTGGT
	FLAG-XhoI-R	TTTCTCGAGCTATTTATCGTCATCATCTTTGTAGTCCTTGTC
p416GAL-Nop58(1–447)-FLAG	NOP58-BHI-F	TTGGATCCATGGCTTACGTTTTAACTGAAACTTC
	NOP58-447-nostop-XhoI-R	TTTCTCGAGTTCATCATCAGAATCGGATTCAGAATC
p416GAL-Nop58(285–447)-FLAG	NOP58-853-BamHI-F	TTGGATCCATGCCAAACTTGACTCAGTTGGTTG
	NOP58-447-nostop-XhoI-R	TTTCTCGAGTTCATCATCAGAATCGGATTCAGAATC
p416GAL-Nop58(345–447)-FLAG	NOP58-1033-BamHI-F	TTGGATCCATGGCCTCTCTTGTTGGTCAAGCTACTGGT
	NOP58-447-nostop-XhoI-R	TTTCTCGAGTTCATCATCAGAATCGGATTCAGAATC
FLAG	FLAG-XhoI-F	TTTCTCGAGATGGATTACAAGGATGACGACGAT
	FLAG-XhoI-noPae-R	TTTCTCGAGAGCTATTTATCGTCATCATCTTTGTAGTCCTTGTC
p416GAL-PIH1	PIH1-BHI-F	TTGGATCCATGGCCGATTTCTTATTGAGACC
	PIH1-XhoI-R	TTTCTCGAGTTATATATATATATATAGTGTGCGT
pRS315-PIH1-GFP-NLS	PIH1-m413-XbaI-F	AAATCTAGAGGAAGGTACCAGTGGCAATTCAGCA
	GFP-NLS-XhoI-R	TTTCTCGAGTTAAACTTTACGTTTCTTCTTTGGAGACTCTTCTTCGGAACCATCAGCAGTACGCTTTTTGTATAGTTCATCCATGCCATGTGTAATCCCAG
pRS315-PIH1-GFP-mNLS	PIH1-m413-XbaI-F	AAATCTAGAGGAAGGTACCAGTGGCAATTCAGCA
	GFP-mNLS-XhoI-R	TTTCTCGAGTTAAACTTTACGTTTAGCCTTTGGAGACTCTTCTTCGGAACCATCAGCAGTGGCTGCTTTGTATAGTTCATCCATGCCATGTGTAATCCCAG
pRS315-PIH1-GFP-NES	PIH1-m413-XbaI-F	AAATCTAGAGGAAGGTACCAGTGGCAATTCAGCA
	GFP-NES-XhoI-R	TTTCTCGAGTTAAGTCTTATTGATGTCTAAACCAGCTAACTTCAAAGCTAAACCTGGAGCTTTGTATAGTTCATCCATGCCATGTGTAATCCCAG
pRS315-PIH1-GFP-mNES	PIH1-m413-XbaI-F	AAATCTAGAGGAAGGTACCAGTGGCAATTCAGCA
	GFP-mNES-XhoI-R	TTTCTCGAGTTAAGTCTTATTAGTGTCAGCACCAGCTAACTTCAAAGCTAAACCTGGAGCTTTGTATAGTTCATCCATGCCATGTGTAATCCCAG

For p416GAL-PIH1 (Figure 2B), the yeast *PIH1* gene was PCR amplified from genomic DNA using oligonucleotides (Table 2) and cloned into *Bam*HI-*Xho*I sites of p416GAL vector. The pYEX-NUP53 and pYEX-NUP53-CΔ plasmids were a kind gift from Dr. Richard W. Wozniak (University of Alberta). To influence the localization of Pih1-GFP four plasmids were constructed: pRS315-PIH1-GFP-NLS, pRS315-PIH1-GFP-mNLS, pRS315-PIH1-GFP-NES, and pRS315-PIH1-GFP-mNES. The DNA sequences were amplified from genomic DNA isolated from PIH1-GFP genomically-tagged strain.

### Protein purification

The construction, expression, and purification of full-length Pih1 and Pih1(1–230) have been described previously [14,26]. The Tah1 and Pih1 proteins were co-expressed using co-expression vector, pCOLADuet-1 transformed into BL21(DE3) pRIL cells. Tah1 had an N-terminal His_6_-tag followed by tobacco etch virus cut site (TEV), HV-tag, while Pih1 was untagged. Protein expression was induced with 1 mM IPTG at 18°C. The co-expressed HV-Tah1/Pih1 complex was purified using Ni-NTA resin, and, subsequently, the N-terminal HV-Tag on Tah1 was removed by incubating with TEV protease. The Tah1-Pih1 complex was further purified on an anion exchange chromatography column (Mono Q). HV-Rvb1 and untagged Rvb2 were also co-expressed using pCOLADuet-1 in BL21(DE3) pRIL, and purified using Ni-NTA resin column. The N-terminal HV-tag of Rvb1 was removed by TEV protease as described above. Subsequently, the Rvb1/Rvb2 complex was purified on Superdex 200 size exclusion column.

### FLAG pulldown assays

FLAG pulldown assays were performed essentially as described [14,57]. C-terminal 3xFLAG-tagged strains were grown in YPD medium to log phase (OD_600_ = approximately 0.6) or stationary phase (OD_600_ > 5.0) at 30°C. For Figure 1B, soluble cell lysate was treated with DNase I (100 U/mL) and/or RNase A (0.5 mg/mL) at 30°C for 30 min before incubating with anti-FLAG (M2) sepharose beads (Sigma). For Figure 1E, the transformants of each p416GAL construct were grown on SD-Ura plates for 2 days at 30°C and incubated in SG (galactose)-Ura medium until log phase for 6 h at 30°C. Cell pellets were disrupted with dry ice using a coffee grinder and dissolved in buffer H [25 mM HEPES-KOH, pH 7.6, 1 mM EDTA, 10% glycerol, 0.02% NP-40, 2.5 mM DTT, 2 mM MgCl_2_, and protease inhibitor cocktail tablets (Roche Diagnostics)] containing 0.1 M KCl. The total cell lysate was clarified by centrifugation and then the supernatant was used for immunoprecipitation using anti-FLAG beads. The immunopurified protein complexes were washed with buffer H containing 0.3 M or 0.5 M KCl. 1 mg/mL FLAG peptide (Sigma) in 0.1 M KCl was used for elution. For Figure 4, the complexes on the α-FLAG beads were further incubated in the absence or presence of 4 mM nucleotides at 30°C for 30 min, and the supernatants were removed. The beads were washed with buffer H containing 0.1 M KCl. The levels of the proteins in each fraction were determined by Western blotting or silver staining.

### GST pulldown assays

Nop58 protein fragments with N-terminal GST-tag were expressed in *E. coli* BL21(DE3) pRIL cells. The fusion proteins were purified on glutathione Sepharose 4B beads following manufacturer’s protocol (GE Healthcare). Pih1 and Pih1(1–230) were purified using the same protocol as previously described [14]. The immobilized GST-Nop58 variants were incubated with Pih1 or Pih1(1–230) for 40 min at 30°C in GST lysis buffer (1×PBS, 0.1% NP40, 10% glycerol, and 1 mM DTT). The beads were washed three times with GST lysis buffer containing 0.5 M NaCl and then rinsed with the GST lysis buffer. The retained proteins were separated on SDS-PAGE gels and visualized by Ponceau S staining and Western blotting. For *in vitro* reconstitution of Nop58 C-terminal domain with R2TP complex (Figure 4D), MagneGST Glutathione Particles (Promega) were used for purification of GST alone and GST-Nop58-C proteins (Figure 1D, upper panel). Then, 0.1 μM of preformed Rvb1/2 or R2TP complex purified from *E. coli* was incubated with the purified glutathione beads-bound proteins at 4°C for 16 h, and the unbound R2TP was washed away using buffer H containing 0.1 M KCl at 23°C.

### Northern blot analysis

Total RNA extraction and Northern analysis were performed as described [14]. Briefly, total RNA from log phase cells and co-immunoprecipitated RNA from FLAG pulldowns were extracted by phenol/chloroform extraction and precipitated by ethanol with 0.5 M LiCl. For snoRNA Northern analysis, 6 μg and 0.6 μg of total RNA, and RNA isolated from pulldown complexes were separated on 5% polyacrylamide-urea gels and electrotransferred to a Biodyne B membrane (Pall). The transferred membrane was hybridized using 5′-^32^P-labeled oligonucleotide probes for U14 (GCGGTCACCGAGAGTACTAACGA) (Figure 3A).

### RT-PCR

FLAG pulldown complexes eluted with 3x FLAG peptide and input soluble cell lysates for the pulldown were treated with RNase-free DNase I at 37°C for 30 min and then the RNAs were extracted with phenol/chloroform and precipitated by ethanol with 0.5 M LiCl. The first strand cDNA was synthesized using reverse primer for U14, U14-90-R (CGGTCACCGAGAGTACTAACG), dNTP, and reverse transcriptase (SuperScript™ III, Life Technologies) from the purified RNA template. The synthesized cDNAs were amplified by PCR using U14-m60-F (TCTCATGAGATTATCAAATGTGGG) and U14-90R as a primer set to detect U14 pre-snoRNA.

### Western blot analysis

For the analysis of protein accumulation in cells, total crude cell lysate was prepared as previously reported [58]. Proteins resolved by SDS-PAGE were transferred to nitrocellulose membranes (Pall). The membrane blots were blocked with 5% non-fat dried milk and incubated for 2 h or overnight with antibodies. The antibodies used were as follows: Rabbit α-Rvb1, α-Rvb2, α-Pih1, and α-Tah1 [14], mouse monoclonal α-FLAG (Sigma), α-actin (Abcam), α-Nop1 (clone 28 F2, EnCor biotechnology Inc.), and α-Nop58 (clone 34B12, EnCor biotechnology Inc.). Polyclonal rabbit antisera for Snu13 and Nop56 were produced using purified recombinant Snu13 and Nop56 proteins from *E. coli* (EZBiolab Inc.). Goat α-rabbit or goat α-mouse IgG antibodies conjugated with horseradish peroxidise (Biorad) were used as secondary antibodies.

### Microscopy

Cells were mounted on glass slides for image analysis. Images were captured using a DMI 6000B fluorescence microscope (Leica Microsystems) at 63× magnification equipped with a spinning-disk head, an argon laser (Quorum Technologies) and ImagEM charge-coupled device camera (Hamamatsu Photonics). For nuclear staining of live cells, yeast cultures were incubated for 10 min with 6× diluted NucBlue Live Cell Stain (Hoechst33342; Life Technologies) before capturing the image. For quantification of GFP signals in the nucleolus and nucleoplasm shown in Figure 6D, the densitometry of GFP signals of approximately 100 cells were measured using NIH ImageJ. The total GFP signals were measured and set as 100%, and the GFP signals which did not overlap with an area stained by Hoechst33342 were counted as nucleolus. For nuclear import analysis of Figure 7B, the transformant of pYEX-NUP53-CΔ or pYEX-NUP53 was grown in SD-Ura until early-log phase, and then washed with SD-Leu-Ura medium and further incubated in SD-Leu-Ura medium for 3 h.

## Additional files

Additional file 1: Figure S1. Wider view of the subcellular localizations of endogenously GFP-tagged R2TP and box C/D snoRNP proteins in log and stationary phase cells. Cells were stained with Hoechst33342 and then analyzed for GFP or Hoechst33342 fluorescence. The images of DIC, GFP (green), and Hoechst33342 (red), and the merged pictures are shown. White arrowheads show nucleoplasmic GFP signals of Nop1-, Nop56-, and Nop58-GFP. Additional file 2: Figure S2. Wider view of the subcellular localizations of endogenously GFP-tagged Rvb1 and Tah1 in WT and *pih1*Δ log phase cells. Cells were stained with Hoechst33342 and then analyzed for GFP or Hoechst33342 fluorescence. The images of DIC, GFP (green), and Hoechst33342 (red), and the merged pictures are shown. Additional file 3: Figure S3. Wider view of the subcellular localizations of endogenously GFP-tagged Nop1, Nop56, and Nop58 in WT and *pih1*Δ log phase cells. Cells were stained with Hoechst33342 and then analyzed for GFP or Hoechst33342 fluorescence. The images of DIC, GFP (green), and Hoechst33342 (red), and the merged pictures are shown. White arrowheads show nucleoplasmic GFP signals of Nop1-GFP, Nop56-GFP, and Nop58-GFP. Additional file 4: Figure S4. Subcellular localizations in log phase cells of endogenously GFP-tagged R2TP and box C/D proteins in SD media, nutrient-limited media lacking carbon or nitrogen sources, and after rapamycin treatment. The images of DIC and GFP signals from GFP-tagged R2TP and box C/D snoRNP proteins are shown. Additional file 5: Figure S5. Subcellular localizations of endogenously GFP-tagged Rvb1 and Rvb2 in log phase WT cells expressing Nup53-CΔ or Nup53 under *CUP1* promoter. The images of DIC and GFP signals from GFP-tagged Rvb1 and Rvb2 are shown. Additional file 6: Figure S6. A model of a signaling-like pathway based on protein stabilization affecting ribosome biogenesis. The unstable protein Pih1 is stabilized by the Hsp90-Tah1 chaperone complex, while Nop58 is stabilized by the R2TP-Hsp90 complex. This protein stabilization pathway regulates box C/D snoRNP maturation and, hence, ribosome biogenesis.
