# Supplementary figures and images for: Nutritional status modulates box C/D snoRNP biogenesis by regulated subcellular relocalization of the R2TP complex
Source: Genome Biol. 2014 Jul 25;15(7):404. doi: 10.1186/s13059-014-0404-4 (PMC4165372; doi:10.1186/s13059-014-0404-4)

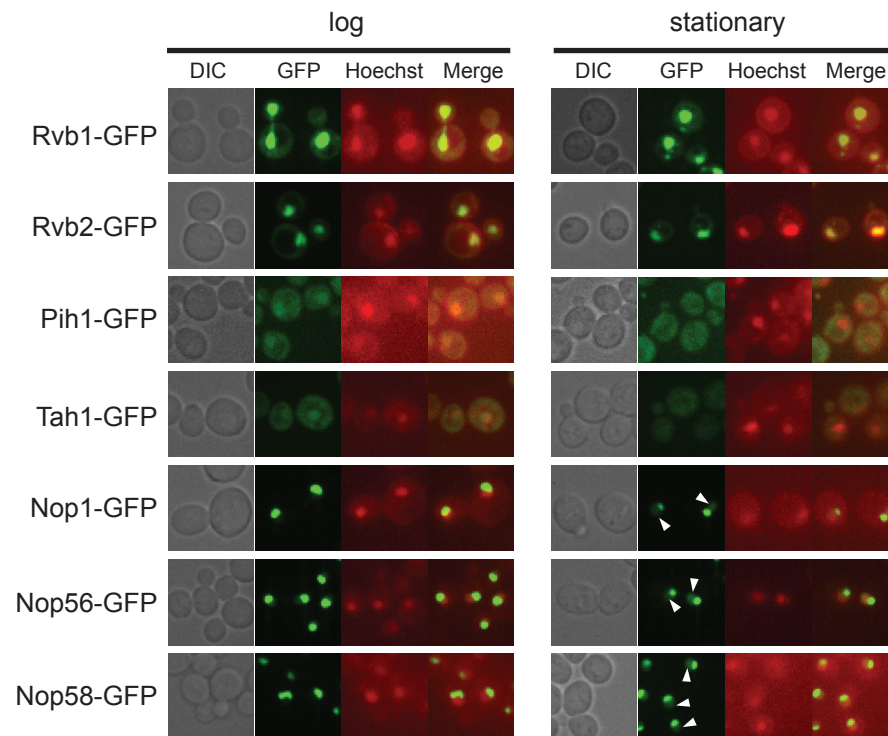

Supplement: Additional file 1: Figure S1. — Wider view of the subcellular localizations of endogenously GFP-tagged R2TP and box C/D snoRNP proteins in log and stationary phase cells. Cells were stained with Hoechst33342 and then analyzed for GFP or Hoechst33342 fluorescence. The images of DIC, GFP (green), and Hoechst33342 (red), and the merged pictures are shown. White arrowheads show nucleoplasmic GFP signals of Nop1-, Nop56-, and Nop58-GFP. [file 13059_2014_404_MOESM1_ESM.pdf]

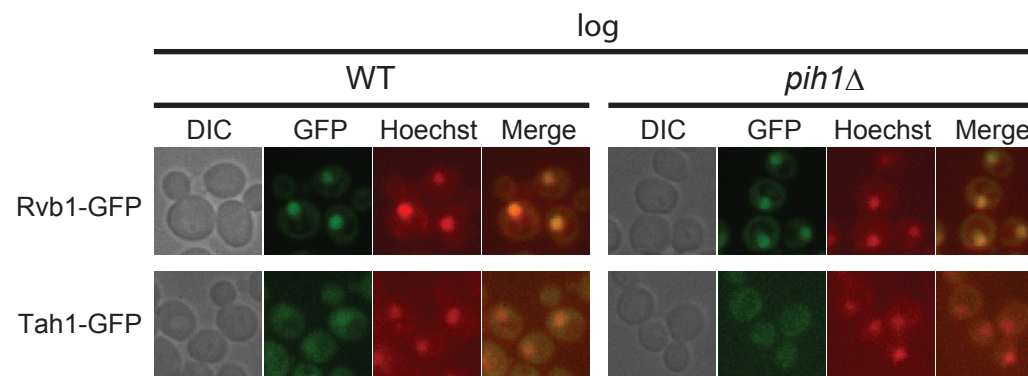

Supplement: Additional file 2: Figure S2. — Wider view of the subcellular localizations of endogenously GFP-tagged Rvb1 and Tah1 in WT and pih1Δ log phase cells. Cells were stained with Hoechst33342 and then analyzed for GFP or Hoechst33342 fluorescence. The images of DIC, GFP (green), and Hoechst33342 (red), and the merged pictures are shown. [file 13059_2014_404_MOESM2_ESM.pdf]

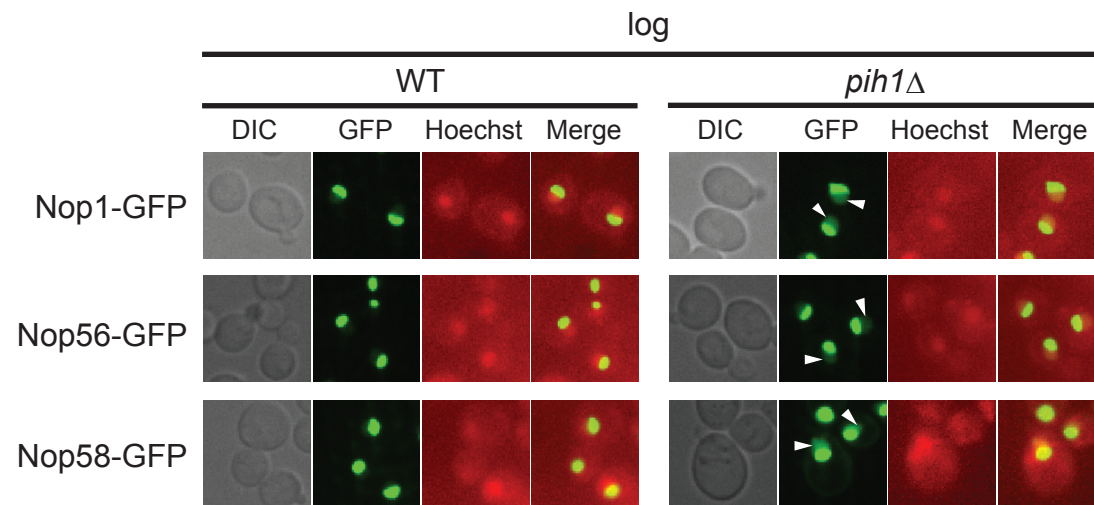

Supplement: Additional file 3: Figure S3. — Wider view of the subcellular localizations of endogenously GFP-tagged Nop1, Nop56, and Nop58 in WT and pih1Δ log phase cells. Cells were stained with Hoechst33342 and then analyzed for GFP or Hoechst33342 fluorescence. The images of DIC, GFP (green), and Hoechst33342 (red), and the merged pictures are shown. White arrowheads show nucleoplasmic GFP signals of Nop1-GFP, Nop56-GFP, and Nop58-GFP. [file 13059_2014_404_MOESM3_ESM.pdf]

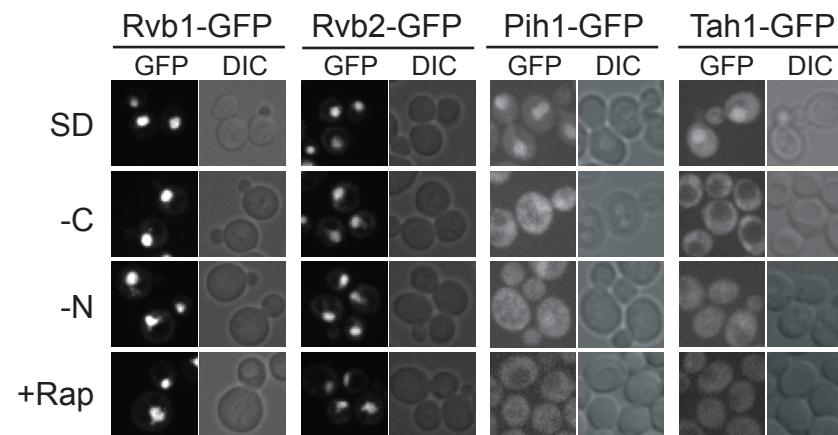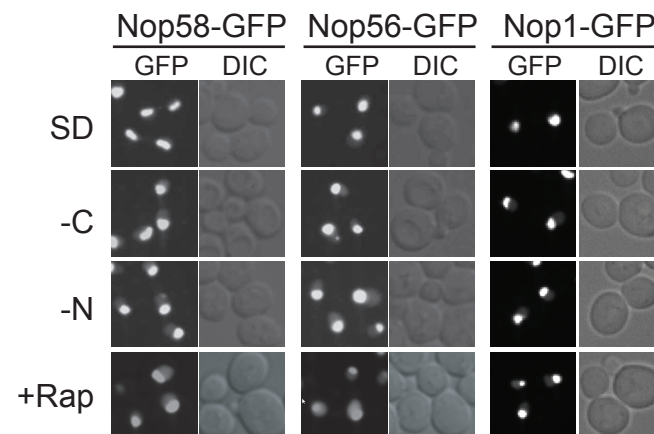

Supplement: Additional file 4: Figure S4. — Subcellular localizations in log phase cells of endogenously GFP-tagged R2TP and box C/D proteins in SD media, nutrient-limited media lacking carbon or nitrogen sources, and after rapamycin treatment. The images of DIC and GFP signals from GFP-tagged R2TP and box C/D snoRNP proteins are shown. [file 13059_2014_404_MOESM4_ESM.pdf]

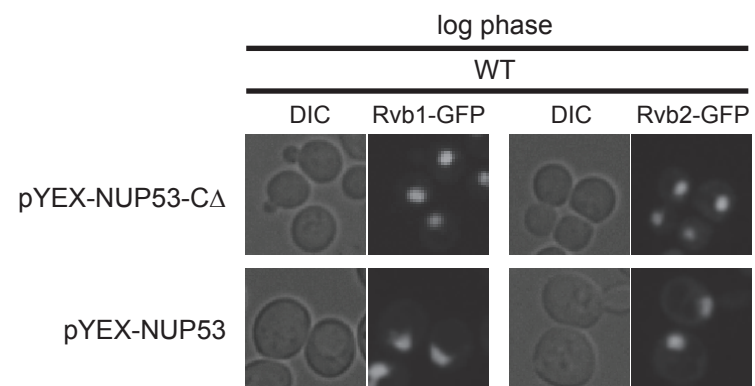

Supplement: Additional file 5: Figure S5. — Subcellular localizations of endogenously GFP-tagged Rvb1 and Rvb2 in log phase WT cells expressing Nup53-CΔ or Nup53 under CUP1 promoter. The images of DIC and GFP signals from GFP-tagged Rvb1 and Rvb2 are shown. [file 13059_2014_404_MOESM5_ESM.pdf]
