# Supplementary material for: Nutritional status modulates box C/D snoRNP biogenesis by regulated subcellular relocalization of the R2TP complex
Source: Genome Biol. 2014 Jul 25;15(7):404. doi: 10.1186/s13059-014-0404-4 (PMC4165372; doi:10.1186/s13059-014-0404-4)
Supplement: Additional file 6: Figure S6. — A model of a signaling-like pathway based on protein stabilization affecting ribosome biogenesis. The unstable protein Pih1 is stabilized by the Hsp90-Tah1 chaperone complex, while Nop58 is stabilized by the R2TP-Hsp90 complex. This protein stabilization pathway regulates box C/D snoRNP maturation and, hence, ribosome biogenesis. [file 13059_2014_404_MOESM6_ESM.pdf]

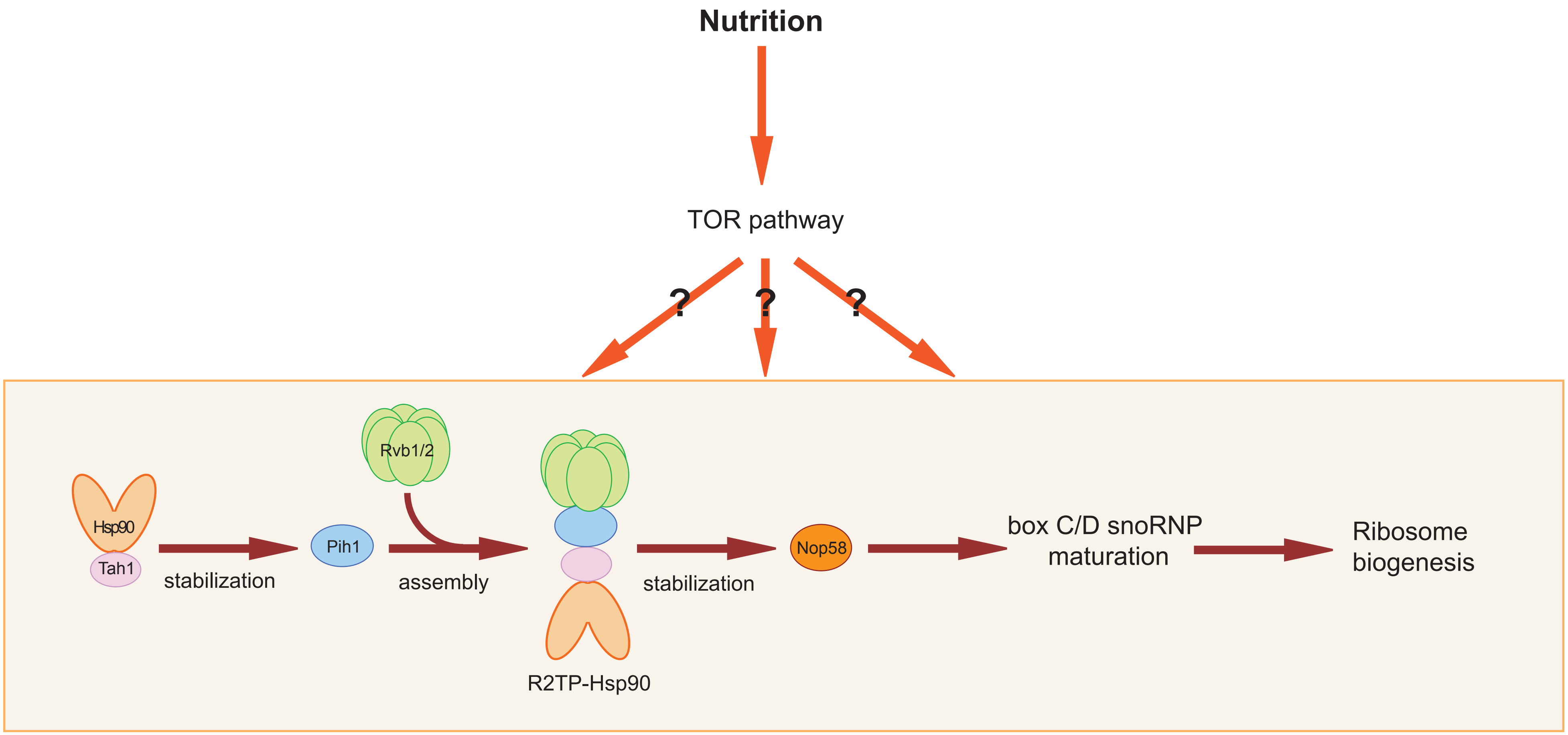
Nutrition
TOR pathway
?
?
?
Rvb1/2
Hsp90
Tah1
stabilization
Pih1
assembly
R2TP-Hsp90
stabilization
Nop58
box C/D snoRNP maturation
Ribosome biogenesis
